# Contrast-enhanced thin-slice abdominal CT with super-resolution deep learning reconstruction technique: evaluation of image quality and visibility of anatomical structures

**DOI:** 10.1007/s11604-024-01685-2

**Published:** 2024-11-14

**Authors:** Atsushi Nakamoto, Hiromitsu Onishi, Takashi Ota, Toru Honda, Takahiro Tsuboyama, Hideyuki Fukui, Kengo Kiso, Shohei Matsumoto, Koki Kaketaka, Takumi Tanigaki, Kei Terashima, Yukihiro Enchi, Shuichi Kawabata, Shinya Nakasone, Mitsuaki Tatsumi, Noriyuki Tomiyama

**Affiliations:** 1 Department of Future Diagnostic Radiology, Osaka University Graduate School of Medicine, 2-2, Yamadaoka, Suita, Osaka 565-0871 Japan; 2 Department of Diagnostic and Interventional Radiology, Osaka University Graduate School of Medicine, 2-2, Yamadaoka, Suita, Osaka 565-0871 Japan; 3 Department of Medical Physics and Engineering, Osaka University Graduate School of Medicine, 1-7, Yamadaoka, Suita, Osaka 565-0871 Japan; 4 Department of Radiology, Kobe University Graduate School of Medicine, 7-5-2 Kusunoki-cho, Chuo-ku, Kobe, Hyogo 650-0017 Japan; 5 Division of Radiology, Department of Medical Technology, Osaka University Hospital, 2-15, Yamadaoka, Suita, Osaka 565-0871 Japan

**Keywords:** Computed tomography, Artificial intelligence, Super-resolution deep learning reconstruction, Iterative reconstruction

## Abstract

**Purpose:**

To compare image quality and visibility of anatomical structures on contrast-enhanced thin-slice abdominal CT images reconstructed using super-resolution deep learning reconstruction (SR-DLR), deep learning-based reconstruction (DLR), and hybrid iterative reconstruction (HIR) algorithms.

**Materials and methods:**

This retrospective study included 54 consecutive patients who underwent contrast-enhanced abdominal CT. Thin-slice images (0.5 mm thickness) were reconstructed using SR-DLR, DLR, and HIR. Objective image noise and contrast-to-noise ratio (CNR) for liver parenchyma relative to muscle were assessed. Two radiologists independently graded image quality using a 5-point rating scale for image noise, sharpness, artifact/blur, and overall image quality. They also graded the visibility of small vessels, main pancreatic duct, ureters, adrenal glands, and right adrenal vein on a 5-point scale.

**Results:**

SR-DLR yielded significantly lower objective image noise and higher CNR than DLR and HIR (*P* < .001). The visual scores of SR-DLR for image noise, sharpness, and overall image quality were significantly higher than those of DLR and HIR for both readers (*P* < .001). Both readers scored significantly higher on SR-DLR than on HIR for visibility for all structures (*P* < .01), and at least one reader scored significantly higher on SR-DLR than on DLR for visibility for all structures (*P* < .05).

**Conclusion:**

SR-DLR reduced image noise and improved image quality of thin-slice abdominal CT images compared to HIR and DLR. This technique is expected to enable further detailed evaluation of small structures.

## Introduction

Recent advances in image reconstruction techniques have significantly improved the image quality of CT. The introduction of iterative reconstruction techniques allowed a significant reduction in image noise compared to the filtered-back projection (FBP) technique, which was the main reconstruction method used until then, leading to a reduction in radiation dose [1]. Today, hybrid iterative reconstruction (HIR) techniques are widely used in the daily clinical practice due to their short reconstruction time.

In recent years, artificial intelligence (AI)-based techniques have been increasingly introduced in diagnostic imaging [2–5]. Deep-learning-based reconstruction (DLR) is an AI-based algorithm for CT image reconstruction that has recently become available for clinical use [6]. Several papers have reported that this algorithm provides further noise reduction and improved image quality compared to HIR [7–13].

Another recent innovation in CT is increased spatial resolution. Ultra-high-resolution CT (UHRCT) has a smaller detector element and X-ray tube focus compared to conventional CT systems, and the images have higher spatial resolution in both in-plane and slice directions [14]. Several previous studies have reported the utility of UHRCT in the abdominopelvic region [15–18]. In particular, thin-slice imaging with UHRCT has been reported to improve the visibility of small anatomical structures such as vessels and ureters [16, 17].

Super-resolution DLR (SR-DLR) is a newly developed AI-based reconstruction technique for CT images that can simultaneously reduce noise and improve spatial resolution [19]. SR-DLR uses the deep convolutional neural network trained with high-resolution, low-noise images obtained by UHRCT as the target data and noisy, low-resolution images simulated from the target data as input data, and this technique allows low-noise, high-resolution images to be reconstructed from noisy, low-resolution data [20]. This technique was originally developed to improve the image quality and resolution of coronary CT, and the usefulness of SR-DLR in coronary CT has been reported in several previous papers [19–24]. However, there have been few reports of its application beyond the coronary arteries [25] and none in the abdominal region. Improved image quality of thin-slice images is important for detailed assessment of anatomical structures and detection of abnormalities in abdominal CT, and SR-DLR is expected to allow delineation of more detailed structures by reducing noise and improving spatial resolution. The portal venous phase is the most commonly acquired image on contrast-enhanced abdominal CT in daily clinical practice, and although 5-mm-thick images are commonly used, the availability of high-quality thin-slice images can improve diagnostic performance when abnormalities are suspected in small structures such as small blood vessels and ureters. The purpose of this study was to compare image quality and visibility of anatomical structures in thin-slice portal venous phase abdominal CT images reconstructed with SR-DLR, DLR, and HIR.

## Materials and methods

### Patients

This retrospective study was approved by our institutional review board, and informed consent was waived. To determine the sample size, a statistical power analysis was performed using G*Power software (latest ver. 3.1.9.7; Heinrich-Heine-Universität Düsseldorf, Germany; http://www.gpower.hhu.de/). Since this study was a multiple group comparison (*n* = 3), α was set at 0.017, power (1-β) was set at 0.8, and effect size was set at 0.5, resulting in a calculated sample size of 47 for the Wilcoxon signed-rank test. Therefore, the target number of cases was set at approximately 50. Fifty-four consecutive patients who underwent contrast-enhanced CT for evaluation of known or suspected abdominal or pelvic disease between March and April 2023 were enrolled (21 men and 33 women; age range, 36–87 years; mean age, 65.2 years). The clinical indications for CT were as follows: follow-up after surgery or radiotherapy for malignancy, *n* = 32; evaluation after chemotherapy for malignancy, *n* = 13; staging for malignancy, *n* = 6; other, *n* = 3.

### CT examination

CT was performed with a 320-slice scanner (AquilionONE GENESIS Edition, Canon Medical Systems, Otawara, Japan). A dose of 2.0 mL/kg body weight of 300 mgI/mL nonionic contrast material was administered intravenously for 50 s using a power injector (Dual Shot GX, Nemoto Kyorindo, Tokyo, Japan), and portal venous phase images were acquired with a scan delay of 90 s. The tube current was individually adjusted using an automatic tube current modulation system (Volume EC, Canon Medical Systems) with a standard deviation (SD) setting of 13 for a 5 mm slice thickness, which is used for abdominal CT in daily practice at our institution. The remaining scan parameters were as follows: scan mode, volume mode; tube voltage, 120 kVp; rotation time, 0.5 s; collimation, 320 × 0.5 mm. From the raw data of each patient, images were reconstructed using SR-DLR (Precise IQ Engine [PIQE], Canon Medical Systems, Cardiac standard), DLR (Advanced intelligent Clear-IQ Engine [AiCE], Canon Medical Systems, BodySharp standard), and HIR (Adaptive Iterative Dose Reduction 3-dimensional [AIDR 3D], Canon Medical Systems, Enhanced standard) with a slice thickness/interval of 0.5/0.5 mm and a field of view of 345 × 345 mm. The matrix size was 512 × 512 for all images. The volume CT dose index (CTDI_vol_) and size-specific dose estimates (SSDE) were recorded with reference to the dose reports.

### Quantitative analysis

A radiologist experienced in abdominal radiology (21 years of experience) placed approximately 100 mm^2^ oval regions of interest (ROIs) on the liver parenchyma, erector spinae muscle, and subcutaneous fat at the level of the liver hilum. ROIs were placed on the HIR images and then copied and pasted onto the DLR and SR-DLR images. ROI placement was repeated three times for each region and the values were averaged. The SD of the CT number of subcutaneous fat was used as an index of image noise. The contrast between liver parenchyma and muscle and the contrast-to-noise ratio (CNR) of liver parenchyma were calculated using the following equations:

$${\text{Contrast}}\, = \,\left| {CT_{liver} - CT_{muscle} } \right|$$

$${\text{CNR}}\, = \,Contrast/SD_{fat}$$

where CT_liver_ and CT_muscle_ are the CT numbers of liver parenchyma and muscle, respectively. SD_fat_ is the SD of the CT number of subcutaneous fat. Measurements were performed with a commercially available workstation (SYNAPSE VINCENT version 5.3.001, FUJIFILM, Tokyo, Japan).

### Qualitative analysis

The three thin-slice images series (HIR, DLR, and SR-DLR) from each patient were randomized into three data sets for subjective image quality assessment, and the order of patients was also randomized. Two radiologists familiar with abdominal radiology (14 and 9 years of experience) independently reviewed the images and rated the image quality in terms of noise level, sharpness, artifact/blur, and overall image quality. Noise level was rated on a 5-point scale: 1, unacceptable image noise; 2, slightly excessive noise; 3, moderate noise; 4, slightly less noise; and 5, minimal noise. Sharpness was assessed by the sharpness of abdominal visceral structures, particularly the liver, using a 5-point scale: 1, extremely blunt edges; 2, slightly blunt edges; 3, moderate sharpness; 4, slightly sharper edges; and 5, very sharper edges. Pixelated blotchy artifacts, often seen in HIR with a high-strength setting or in model-based iterative reconstruction (MBIR) images [26–28], and blur, reportedly seen in DLR with a high-strength setting [29], were scored on a 5-point scale: 1, unacceptable artifacts and/or blur; 2, slightly excessive artifacts and/or blur; 3, moderate artifacts and/or blur; 4, slight artifacts and/or blur; and 5, no artifacts or blur. Overall image quality was rated on a 5-point scale: 1, poor; 2, fair; 3, moderate; 4, good; and 5, excellent. Visibility of small vessels, main pancreatic duct, ureter, adrenal gland, and right adrenal vein was also rated on a 5-point scale. The criteria for each score in the visibility evaluation are listed in Table 1. Qualitative analyses were performed using a commercially available workstation (SYNAPSE VINCENT version 5.3.001, FUJIFILM). To minimize the recall bias, evaluation of each dataset was separated by a time interval of at least 2 weeks.

**Table 1:** Criteria for each score in the visibility assessment of anatomical structures

	Score	Criteria
Small vessels	1	PHA is partially unclear
	2	PHA is clear, but proximal RHA/LHA is mostly unclear
	3	PHA is clear, but proximal RHA/LHA is partially unclear
	4	PHA to proximal RHA/LHA is clear, but distal RHA/LHA is partially unclear
	5	PHA to distal RHA/LHA is clearly delineated
Main pancreatic duct	1	Invisible in most areas
	2	Partially visible
	3	Visible about halfway
	4	Mostly visible
	5	Fully visible
Ureters	1	More than half unclear on both sides
	2	More than half unclear on one side
	3	About one-third unclear on at least one side
	4	Partially unclear on at least one side
	5	Fully visible on both sides
Adrenal glands	1	Margins are mostly indistinct bilaterally
	2	Margins are mostly indistinct on one side
	3	Margins about half indistinct on at least one side
	4	Margins partially indistinct on at least one side
	5	Margins completely clear bilaterally
Right adrenal vein	1	Invisible
	2	Probably not visible
	3	Equivocal
	4	Probably visible
	5	Visible

*PHA* proper hepatic artery, *RHA* right hepatic artery. *LHA* left hepatic artery

### Statistical analysis

CT number of liver and muscle, contrast between liver and muscle, objective image noise, and CNR of the liver parenchyma were compared among HIR, DLR, and SR-DLR using Friedman’s test followed by post hoc Wilcoxon signed-rank test with Bonferroni correction (*n* = 3). Visual scores of the qualitative analyses were also compared using Friedman’s test followed by post hoc Wilcoxon signed-rank test with Bonferroni correction (*n* = 3). The weighted kappa statistic was used to assess inter-reader agreement. All statistical analyses were performed using R (version 4.2.2, R Foundation for Statistical Computing, Vienna, Austria). For all tests, a *P* value < 0.05 was considered significant.

## Results

### Quantitative analysis

The mean ± SD of CTDI_vol_ and SSDE for the CT examination were 6.31 ± 1.97 and 9.07 ± 2.12 mGy, respectively. CT number of liver parenchyma and muscle, contrast between liver and muscle, objective image noise, and CNR of liver parenchyma for each reconstruction algorithm are summarized in Table 2. CT number of liver parenchyma and muscle were higher in the order of SR-DLR, HIR, and DLR, with significant differences among them (*P* < 0.001). Contrast between liver and muscle was significantly higher for DLR compared to HIR and SR-DLR (*P* < 0.001). Objective image noise was significantly lower in DLR compared to HIR and even significantly lower in SR-DLR (*P* < 0.001). CNR of liver parenchyma was significantly higher in DLR compared to HIR, and even significantly higher in SR-DLR (*P* < 0.001).

**Table 2:** CT number of liver and muscle, contrast between liver and muscle, objective image noise, and CNR of liver parenchyma for HIR, DLR, and SR-DLR

				*P* value		
	HIR	DLR	SR-DLR	HIR vs DLR	HIR vs SR-DLR	DLR vs SR-DLR
CT number of liver (HU)	111.7 (99.4–119.0)	111.4 (98.9–118.2)	113.9 (101.4–121.2)	** < 0.001**	** < 0.001**	** < 0.001**
CT number of muscle (HU)	70.6 (64.7–73.3)	68.7 (64.0–71.7)	73.6 (69.4–77.4)	** < 0.001**	** < 0.001**	** < 0.001**
Contrast between liver and muscle (HU)	41.2 (30.6–47.0)	42.1 (31.1–49.9)	41.2 (30.8–48.6)	** < 0.001**	> 0.999	** < 0.001**
Image noise (HU)	14.1 (12.8–15.3)	12.8 (11.7–13.6)	9.5 (8.8–11.6)	** < 0.001**	** < 0.001**	** < 0.001**
CNR of liver	2.9 (2.1–3.4)	3.3 (2.4–3.8)	4.3 (2.8–4.9)	** < 0.001**	** < 0.001**	** < 0.001**

Data are median (interquartile range)

*P* values for comparisons that were statistically significant are in bold

*HIR* hybrid iterative reconstruction, *DLR* deep-learning-based reconstruction, *SR-DLR* super-resolution deep learning reconstruction, *CNR* contrast to noise ratio

### Qualitative analysis

**Fig. 1:**
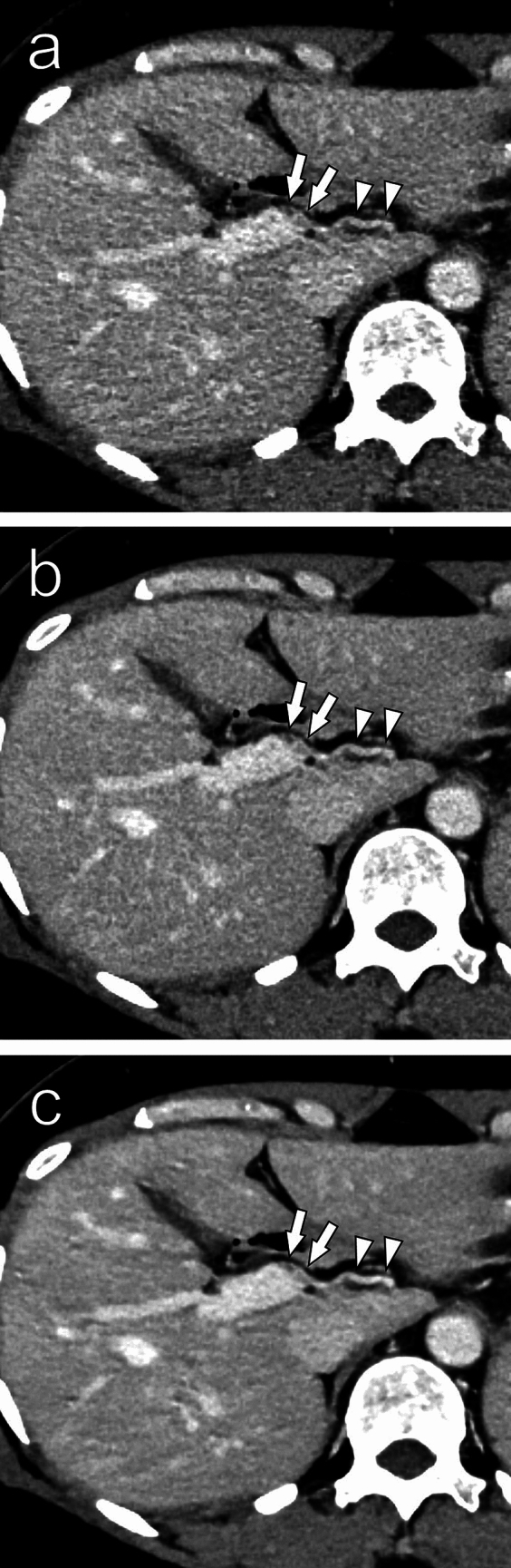
Thin-slice contrast-enhanced images of a male in his 60 s reconstructed with HIR (**a**), DLR (**b**), and SR-DLR (**c**). Noise is reduced in the SR-DLR image compared to the HIR and DLR images. The right hepatic artery (arrows) and the left hepatic artery (arrowheads) are better visualized in the SR-DLR image (**c**) than in the HIR (**a**) and DLR (**b**) images

Table 3 shows the mean visual scores for each reconstruction algorithm. In both readers, the SR-DLR images scored significantly higher than the DLR and HIR images in terms of subjective image noise, sharpness, and overall image quality (*P* < 0.001) (Fig. 1). Artifacts and blur were not observed in almost all cases, and there were no significant differences in the scores.

**Table 3:** Visual scores of qualitative analyses

				*P* value		
	HIR	DLR	SR-DLR	HIR vs DLR	HIR vs SR-DLR	DLR vs SR-DLR
Reader 1						
Subjective image noise	3 (2–3)	4 (3–4)	4 (4–4)	** < 0.001**	** < 0.001**	** < 0.001**
Sharpness	3 (2–3)	4 (3–4)	4 (4–4)	** < 0.001**	** < 0.001**	** < 0.001**
Artifact/blur	5 (5–5)	5 (5–5)	5 (5–5)	> 0.999	> 0.999	> 0.999
Overall image quality	3 (2–3)	4 (3–4)	4 (4–4)	** < 0.001**	** < 0.001**	** < 0.001**
Reader 2						
Subjective image noise	2 (2–2)	3 (3–4)	5 (4–5)	** < 0.001**	** < 0.001**	** < 0.001**
Sharpness	2 (2–2)	4 (3–4)	5 (4–5)	** < 0.001**	** < 0.001**	** < 0.001**
Artifact/blur	5 (5–5)	5 (5–5)	5 (5–5)	> 0.999	> 0.999	> 0.999
Overall image quality	2 (2–2)	3 (3–4)	5 (4–5)	** < 0.001**	** < 0.001**	** < 0.001**

Data are median (interquartile range)

*P* values for comparisons that were statistically significant are in bold

*HIR* hybrid iterative reconstruction, *DLR* deep-learning-based reconstruction, *SR-DLR* super-resolution deep learning reconstruction

The weighted kappa coefficients were 0.65 (substantial agreement) for image noise, 0.67 (substantial agreement) for sharpness, − 0.01 (no agreement) for artifact/blur, and 0.65 (substantial agreement) for overall image quality, respectively.

### Visibility of small structures

**Fig. 2:**
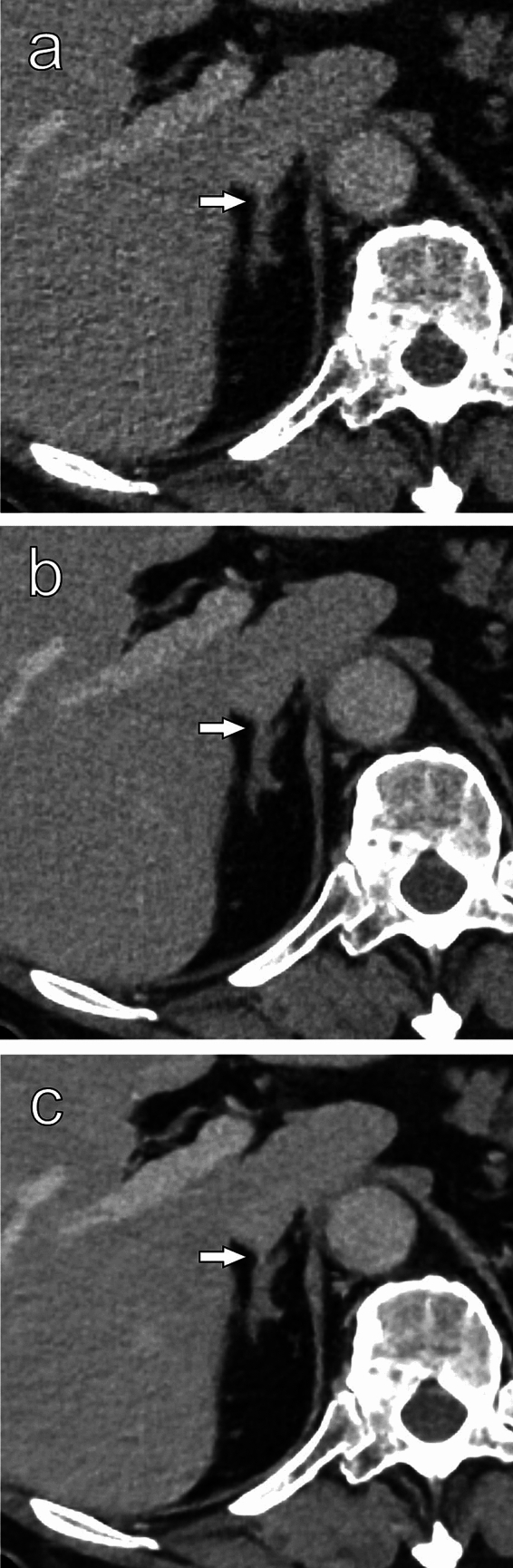
Thin-slice contrast-enhanced images of a male in his 50 s reconstructed with HIR (**a**), DLR (**b**), and SR-DLR (**c**). The boundary between the right adrenal gland and the surrounding tissue is sharpest in the SR-DLR image (**c**). The right adrenal vein (arrow) is best visualized in the SR-DLR image (**c**)

**Fig. 3:**
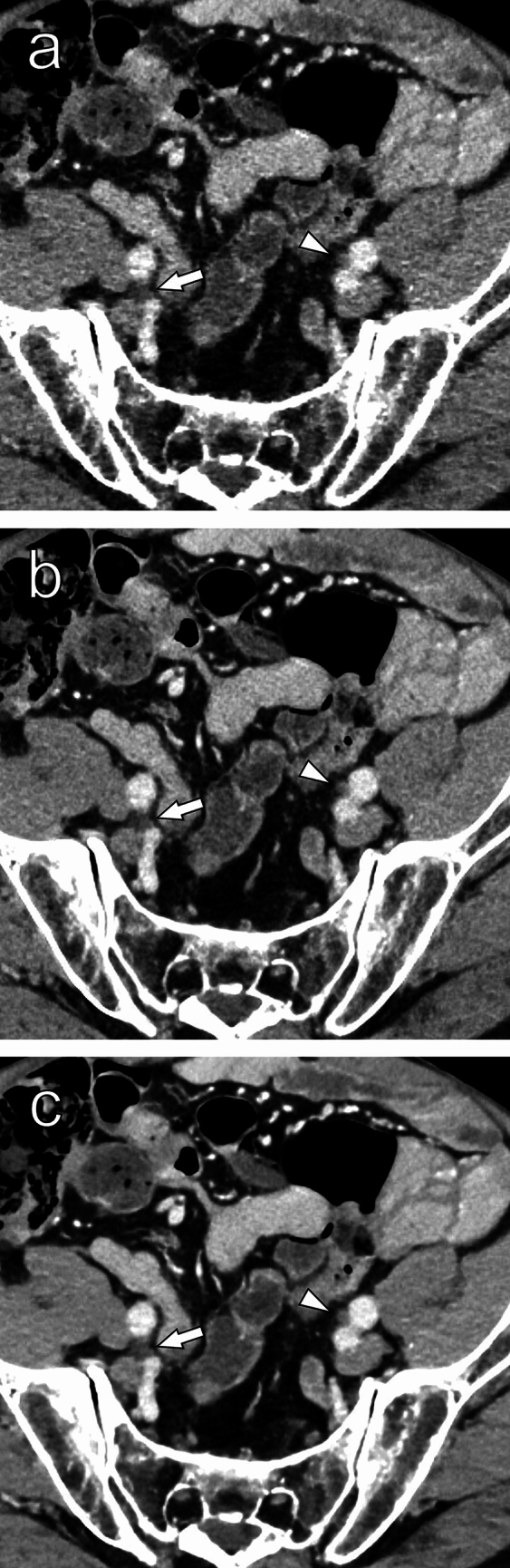
Thin-slice contrast-enhanced images of a female in her 40 s reconstructed with HIR (**a**), DLR (**b**), and SR-DLR (**c**). Although the border between the right ureter (arrow) and surrounding structures is slightly obscured in the HIR image (**a**), the border is more clearly seen in the SR-DLR image (**c**). The left ureter (arrowhead) can also be seen more clearly in the SR-DLR image (**c**). Other organs, including the wall of the small intestine, are also more clearly delineated in the SR-DLR image

Table 4 summarizes the scores for visibility of anatomical structures. For all structures, both readers’ scores were significantly higher on SR-DLR than on HIR (*P* < 0.05). Visibility of small vessels and adrenal glands was significantly higher on SR-DLR compared to DLR by both readers, and main pancreatic duct, ureter, and right adrenal vein were significantly higher by one reader (*P* < 0.05) (Fig. 2, Fig. 3).

**Table 4:** Visibility scores for abdominal structures

				*P* value		
	HIR	DLR	SR-DLR	HIR vs DLR	HIR vs SR-DLR	DLR vs SR-DLR
Reader 1						
Small vessels	4 (3–4)	4 (4–4)	4 (4–5)	** < 0.001**	** < 0.001**	**0.049**
Main pancreatic duct	3 (1.25–4)	4 (2–4)	4 (1.25–5)	**0.002**	**0.001**	0.528
Ureters	3.5 (3–4)	4 (4–4)	4 (4–5)	**0.002**	** < 0.001**	** < 0.001**
Adrenal glands	4 (4–4.75)	4 (4–5)	5 (4–5)	** < 0.001**	** < 0.001**	**0.029**
Right adrenal vein	3 (1–3)	4 (1–4)	4 (1.25–5)	**0.005**	** < 0.001**	0.119
Reader 2						
Small vessels	4 (4–5)	5 (4–5)	5 (5–5)	** < 0.001**	** < 0.001**	**0.002**
Main pancreatic duct	3 (2–4)	3 (2–4.75)	3.5 (2–5)	0.152	**0.002**	**0.018**
Ureters	4 (3–4)	4 (3–5)	4.5 (3–5)	0.057	** < 0.001**	0.076
Adrenal glands	5 (5–5)	5 (5–5)	5 (5–5)	> 0.999	**0.025**	**0.014**
Right adrenal vein	2 (1–3)	3 (1–4)	3 (2–5)	0.681	**0.002**	**0.011**

Data are median (interquartile range)

*P* values for comparisons that were statistically significant are in bold

*HIR* hybrid iterative reconstruction, *DLR* deep-learning-based reconstruction, *SR-DLR* super-resolution deep learning reconstruction

The weighted kappa coefficients were 0.32 (fair agreement) for small vessels, 0.47 (fair agreement) for main pancreatic duct, 0.38 (fair agreement) for ureter, 0.18 (slight agreement) for adrenal glands, and 0.58 (moderate agreement) for right adrenal vein, respectively.

## Discussion

Our results demonstrated that thin-slice abdominal CT images reconstructed with SR-DLR resulted in significantly lower objective image noise and higher CNR compared to those reconstructed with HIR or DLR. While many previous studies have reported reduced noise in CT images with DLR compared to HIR [7–12, 29], the current results suggest that the use of SR-DLR can further reduce noise and improve image quality. This is consistent with several previous reports investigating the utility of SR-DLR in coronary CT [19, 21, 23]. CT numbers of liver and muscle were higher with SR-DLR compared to HIR, and the difference averaged approximately 3 HU. The cause of this difference is not clear, and one reason may be that SR-DLR is not optimized for abdominal CT. CT numbers tended to be higher overall, and no difference in contrast between liver and muscle was observed between HIR and SR-DLR, suggesting that lesion contrast is not expected to be significantly affected.

In the visual evaluation, SR-DLR scored significantly higher than DLR and HIR in noise, sharpness, and overall image quality. Therefore, the visual evaluation also shows that SR-DLR provides further noise reduction and image quality improvement compared to DLR. It has been reported that images reconstructed using HIR or MBIR with a high-strength setting can have a characteristic texture, called a blotchy appearance [26–28]. In addition, it has been reported that mild blurring can be observed in DLR images at the high-strength setting [29]. In the current study, neither of these findings were observed in SR-DLR images in almost all cases, suggesting that SR-DLR can reduce noise while preserving image texture, resulting in a high score for overall image quality. Only the standard setting was evaluated in the current study, and further studies would be needed to investigate the image textures with SR-DLR at higher settings.

Thin-slice abdominal CT images are useful for evaluation of detailed anatomic structures, such as evaluation of anatomic variations in small vessels, detection of vascular lesions, and evaluation of the right adrenal vein prior to adrenal venous sampling [30–33]. Our results showed that SR-DLR provided significantly improved visibility compared to HIR in all anatomical structures evaluated. In addition, SR-DLR scored higher than DLR in all anatomical structures, with significant differences in at least one reader. This could be attributed to the fact that SR-DLR provided further noise reduction, making it easier to recognize the boundaries with surrounding tissues. In addition, the increased spatial resolution provided by SR-DLR may have resulted in further improvements in visibility compared to DLR. Kaga et al. reported that the visibility of anatomical structures on unenhanced low-dose abdominal CT images reconstructed with DLR was comparable to or better than that on standard-dose images reconstructed with HIR [8]. As with the low-dose CT images, the effect of DLR on improving image quality would be significant in thin-slice images due to the large amount of noise. Therefore, SR-DLR, which provides greater noise reduction and improved spatial resolution, is a promising technique for improving the visibility of anatomical structures in thin-slice abdominal CT.

This study had several limitations. First, the SR-DLR algorithm used in the current study was developed for coronary CT and is not optimized for abdominal CT. This algorithm can only be applied in volume scan mode and does not support helical scan mode. In addition, the current SR-DLR can only reconstruct thin-slice images, and 5-mm-thick images must be reformatted from thin-slice images. Since abdominal CT is usually acquired using the helical scan mode and interpreted with 5-mm-thick images, it is necessary for SR-DLR to be modified to support helical scan mode and thicker slice thickness reconstruction. Second, we evaluated only the visibility of anatomical structures and did not investigate the lesion detectability. Given the improved visibility of anatomical structures, it is expected that SR-DLR would similarly improve the diagnostic performance of lesions, although further studies are needed to confirm this. Third, this study investigated only images obtained with the standard-dose setting and did not examine its usefulness in low-dose CT. Further studies are needed to assess how much dose reduction can be achieved using SR-DLR. Finally, the kappa coefficients were low for some ratings. However, we attribute this to a bias toward higher or lower scores. For example, the kappa coefficient for the artifact/blur rating was -0.01, while two readers’ scores were mostly 5, a 99% agreement.

In conclusion, SR-DLR reduced image noise and improved the image quality of portal venous phase thin-slice abdominal CT images compared with HIR and DLR. This technique is expected to enable further detailed evaluation of small abdominal structures.
